# Nasal TRPA1 mediates irritant‐induced bradypnea in mice

**DOI:** 10.14814/phy2.13098

**Published:** 2016-12-30

**Authors:** Keiichi Inui, ChangPing Chen, Jordan L. Pauli, Chiharu Kuroki, Shogo Tashiro, Yuichi Kanmura, Hideki Kashiwadani, Tomoyuki Kuwaki

**Affiliations:** ^1^ Department of PhysiologyKagoshima University Graduate School of Medical & Dental SciencesKagoshimaJapan; ^2^ Anesthesiology & Critical Care MedicineKagoshima University Graduate School of Medical & Dental SciencesKagoshimaJapan

**Keywords:** Allyl isothiocyanate, extracellular signal‐regulated kinase, olfactory nerve, trigeminal nerve, vagal nerve

## Abstract

Transient receptor potential ankyrin 1 (TRPA1), a member of the TRP superfamily, exists in sensory neurons such as trigeminal neurons innervating the nasal cavity and vagal neurons innervating the trachea and the lung. Although TRPA1 has been proposed as an irritant receptor that, when stimulated, triggers bradypnea, precise locations of the receptors responsible have not been elucidated. Here, we examined the relative importance of TRPA1 located in the upper airway (nasal) and the lower airway (trachea/lungs) in urethane‐anesthetized mice. To stimulate the upper and lower airways separately, two cannulas were inserted through a hole made in the trachea just caudal to the thyroid cartilage, one into the nasal cavity and the second into the lower trachea. A vapor of one of the TRPA1‐agonists, allyl isothiocyanate (AITC), was introduced by placing a piece of cotton paper soaked with AITC solution into the airline. AITC decreased the respiratory frequency when applied to the upper airway (ca −30%) but not to the lower airway (ca −5%). No response was observed in TRPA1 knockout mice. Contribution of the olfactory nerve seemed minimal because olfactory bulbectomized wild‐type mice showed a similar response to that of the intact mice. AITC‐induced bradypnea seemed to be mediated, at least in part, by the trigeminal nerve because trigeminal ganglion neurons were activated by AITC as revealed by an increase in the phosphorylated form of extracellular signal‐regulated kinase in the neurons. These data clearly show that trigeminal TRPA1 in the nasal cavity play an essential role in irritant‐induced bradypnea.

## Introduction

Defense against harmful airborne materials such as reactive chemicals, toxic particulates, and infectious agents is essential for animals to protect their lungs and life. The defense mechanisms include: an acute expelling strategy via coughing and sneezing, a stopping strategy via airway contraction and bradypnea, and a relatively slow repelling strategy via mucus secretion and inflammation. These defensive responses are initiated by peripheral chemosensory nerve endings that project into the airway lining. The nerve endings in the nasal cavity are part of the trigeminal nerve, and the nerve endings in the trachea and lungs are part of the vagus nerve. Although both the trigeminal and vagus nerves play roles as chemosensors, the resultant effects of their activation may be distinct and, therefore, may be functionally differentiated.

Irritant‐induced bradypnea, the acute decrease in respiratory rates following chemical exposure, is a defense mechanism that is commonly observed among species even when anesthetized (Bessac and Jordt 2008). In addition, bradypnea in mice has been established as the standard method for determining acceptable exposure levels for general public health (Kuwabara et al. 2007). Although trigeminal contribution to the irritant‐induced bradypnea has long been known and widely accepted (Ulrich et al. 1972; Vijayaraghavan et al. 1993), the possibility of vagal contribution has also been reported (Coleridge and Coleridge 1986; Prabhakar et al. 1986; Kou et al. 1995; Wang et al. 1996; Nassenstein et al. 2008). Therefore, the functional differentiation of the trigeminal and vagal nerves and their relative importance in regard to irritant‐induced bradypnea is still an open question.

Recent studies have identified transient receptor potential ankyrin 1 (TRPA1), a member of the TRP superfamily, as a candidate for a long unknown irritant receptor in the airway (Nassenstein et al. 2008; Bessac et al. 2008; Grace et al., 2014). In early 2008, Nassenstein et al. reported TRPA1 expression in vagal afferent nerves innervating mouse lungs by single‐cell RT‐PCR (Nassenstein et al. 2008). They showed cinnamaldehyde, a TRPA1 agonist, that evoked action potentials in vagal C‐fibers. Aerosol of cinnamaldehyde also induced bradypnea in vivo. In the same year, Bessac et al. (2008) described oxidant (OCl^−^ or H_2_O_2_)‐induced activation (calcium influx) in TRPA1‐expressing cell lines and cultured trigeminal and afferent vagal (nodose) ganglia cells in vitro. They also showed oxidant‐induced bradypnea in vivo, which was severely blunted in the TRPA1 knockout mice. Other groups, including ours, have also shown TRPA1 expression in the trigeminal and nodose ganglia neurons and TRPA1 agonist‐induced bradypnea (Kim et al. 2010; Takahashi et al. 2011; Biringerova et al. 2013; Yonemitsu et al. 2013). Although these studies clearly showed an essential role of TRPA1 in airborne irritant‐induced bradypnea, location (trigeminal, vagal, or both) of the responsible TRPA1 in vivo remained unclear.

In this study, we examined the relative importance of TRPA1 located in the upper airway (nasal) and the lower airway (trachea/lungs) on irritant‐induced bradypnea in urethane‐anesthetized mice. Contribution of the olfactory nerves was also examined. In the olfactory bulb, the first relay of olfactory sensation, distributions of trigeminal branches (Schafer et al. 2002), and the presence of TRPA1 mRNA (Dong et al. 2012) have been reported. Olfactory sensation may modulate some of the defensive responses because certain pungent chemicals also have specific smells and the trigeminal system has been shown to modulate recognition of those odors (Cain and Murphy 1980; Jacquot et al. 2004). We found that nasal TRPA1 was essential for irritant‐induced bradypnea; so, next we examined possible activation of the trigeminal neurons using the phosphorylated form of extracellular signal‐regulated kinase (pERK) as the activation marker to confirm contribution of the trigeminal system in irritant‐induced bradypnea.

## Materials and Methods

### Ethical approval

All experimental procedures were performed in accordance with the guiding principles for the care and use of animals in the field of physiological sciences published by the Physiological Society of Japan (2015) and approved by the Institutional Animal Use Committees at Kagoshima University (MD13007).

### Animals

TRPA1 knockout (KO) mice were purchased from the Jackson Laboratory and genotyped as previously described (Kwan et al. 2006). They were maintained as heterozygotes in our facility and crossed to obtain null mutants and wild‐type (WT) littermates (male, 20–30 weeks old). TRPA1 KO mice were backcrossed with C57BL/6 mice (CLEA, Japan) for more than 10 generations and were thought as congenic to C57BL/6. C57BL/6 were also used for olfactory bulbectomy (OB) procedures.

### Experimental setup and measurement of respiration

**Figure 1:**
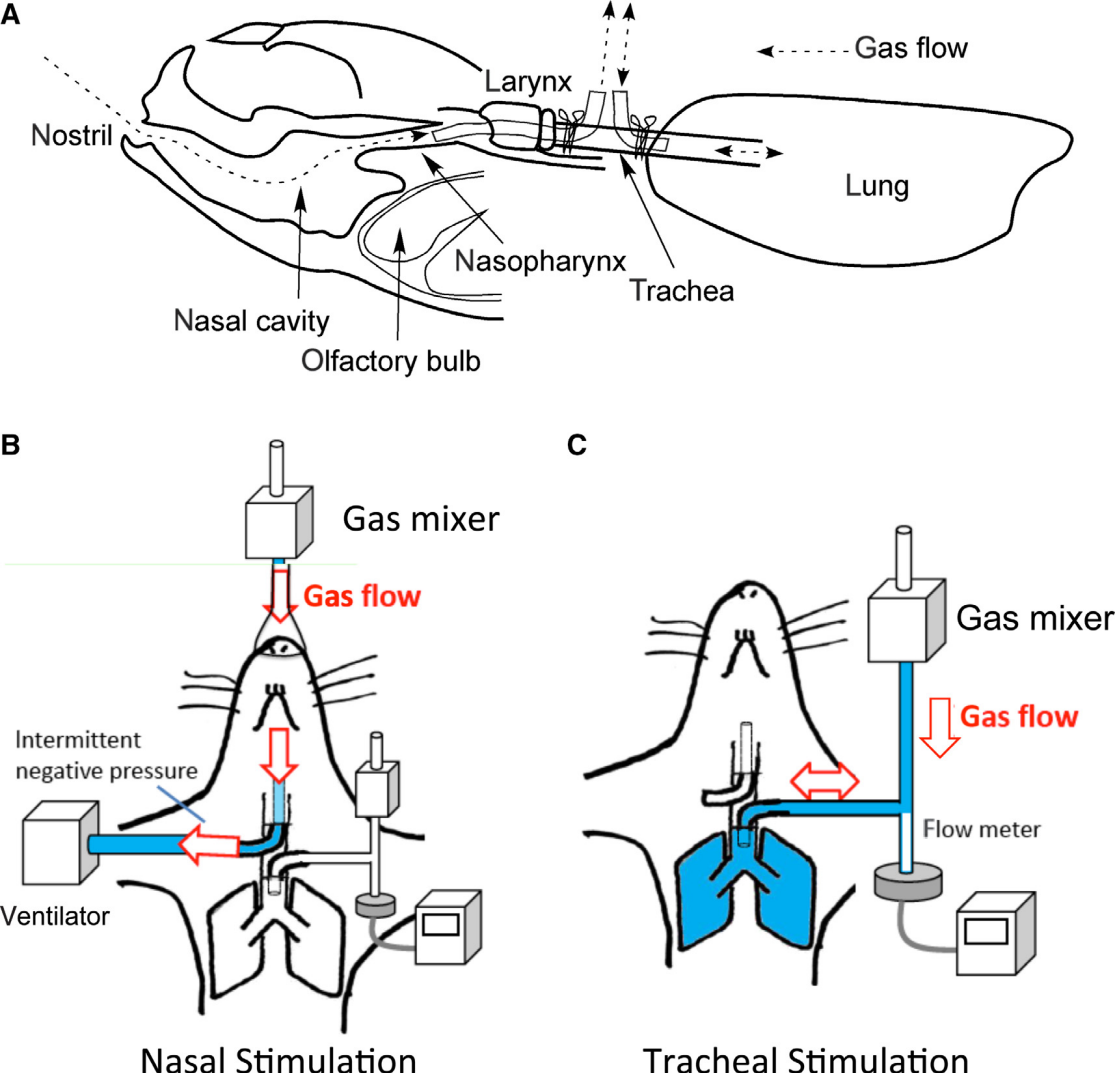
Schematic drawing of the experimental design. (A) Lateral view of a mouse's head and lung showing the position of the two tubes and their relationship to the nasal cavity and trachea. (B, C) Ventral view of the setup showing gas flow during nasal stimulation in (B) and tracheal stimulation in (C).

Mice were anaesthetised with an intraperitoneal injection of urethane (1.3–1.5 g kg^**−**1^). The adequacy of the anesthesia was judged by the absence of the withdrawal reflex to a pinching stimulus. Two polyethylene tubes (outer diameter=1.2 mm, L‐shaped and heat polished to have blunt ends) were inserted into holes cut into the upper trachea (Fig. 1A) so that the nasal cavity and the lower trachea/lungs could be stimulated separately.

The outer end of the upper tube was connected to a ventilator (Mini Vent type 845, Hugo Sachs Elektronik, Germany) and intermittent negative pressure was applied to mimic inspiratory gas flow (stroke volume was set as 200 *μ*L and frequency was 150 min^**−**1^) (Fig. 1B). Proper location of the tip of the upper tube in the nasopharynx was visually confirmed by movement of a small piece of paper that was placed in front of the nostril.

The outer end of the lower tube was connected to a gas source (compressed air tank and air flow was regulated to be 0.6 L/min) and a Lilley‐type flow resistance tube (TV‐241T, Nihon Kohden, Tokyo, Japan) through a t‐tube (Fig. 1B, C). Respiratory flow rate was monitored by a differential pressure transducer (TP602T and AR601G, Nihon Kohden). The animals were allowed to breathe spontaneously.

The concentrations of O_2_ and CO_2_ in the inhalation gas were monitored by an O_2_ sensor (model JKO‐25LJ II CM, JIKCO, Tokyo, Japan) and a CO_2_ monitor (Capstar‐100 Carbon Dioxide Analyzer, CWE Inc., Ardmore, PA), respectively. Body temperature was kept constant at 37 ± 1°C by a heating pad connected to a thermo controller (ATB‐1100, Nihon Kohden) to avoid secondary effects from a possible thermo‐sensitive role of TRPA1 (Kwan et al. 2006; Saito et al. 2014). Room temperature was also kept constant at 25 ± 1°C to achieve reproducible vaporization of AITC. After completing surgical preparations, the animal was allowed to breathe normal room air for over 30 min for stabilization before experimentation.

### Experimental protocols

We examined the possible effects of exposure to a vapor of allyl isothiocyanate (AITC) and 10% CO_2_ (with 20% O_2_ and residual N_2_) on respiratory frequency and tidal volume by applying three stimuli in the following order: AITC for 30 sec, AITC for 60 sec, and CO_2_ for 60 sec. The interstimulus interval was 10 min and began after all respiratory parameters returned to baseline. The order of upper (nasal) and lower (trachea/lungs) stimuli was randomized among the animals. AITC vapor was introduced into the gasline by placing a piece of cotton paper (1 cm x 1 cm) soaked with an AITC solution (20 *μ*L) into the gas‐mixing chamber (Fig. 1B, C).

### Chemicals and determination of gas concentration

An AITC solution (98%) was purchased from Nakarai Chemicals (Tokyo, Japan). The concentration of the AITC vapor was determined using the detector tube method (No.149, Gastec Corp., Ayase, Japan) and was found to be 20 ppm (*n* = 2) at the outlet port of the gas‐mixing chamber at the end of 60 sec of application.

### Olfactory bulbectomy

Bilateral olfactory bulbs were surgically suctioned under ketamine (100 mg/g) and xylazine (10 mg/kg) anesthesia. The mice were treated with an analgesic (0.05 mg kg^**−**1^ buprenorphine, S.C.), the hole drilled through the skull was covered with an ointment containing antibiotic bacitracin and neomycin, and the skin was sutured. The mice were allowed at least 2 weeks of recovery before the experiment began. After the experiment, all the animals were put under heavy anesthesia and euthanized via cervical dislocation, and the brain was visually inspected to confirm proper removal of the olfactory bulbs.

### Immunohistochemical analysis for activation of trigeminal nerve

To examine possible activation of the trigeminal nerve by AITC, we used immunohistochemical detection of pERK in the trigeminal ganglion neurons. pERK is a cellular activation marker that has a more rapid and narrow time window than that of other activation markers including c‐Fos, a popular activation marker (Antoine et al. 2014). For this purpose, urethane‐anesthetized WT, OB, and KO animals (which were different from those used in the respiratory measurement and not intubated) were exposed to the vapor of AITC or water for 60 sec in the nose (see Fig. 1B). After a waiting period of 3 min, the animal was transcardially perfused with 0.01 mol/L phosphate‐buffered saline (PBS) followed by a fixative solution containing 4% paraformaldehyde in PBS. The trigeminal ganglia were dissected and postfixed in the same fixative solution for 24 h at 4°C. After cryoprotection with 30% sucrose in PBS, 16‐*μ*m‐thick serial longitudinal frozen sections were cut and every fourth section (10 slices/animal) was immunohistochemically stained for pERK (1/400, raised in rabbit, #4370S, Cell Signaling Technology) and a neuronal marker, NeuN (1/400, raised in guinea pig, #266004, Synaptic Systems). pERK was visualized with a biotinylated donkey anti‐rabbit IgG antibody (1/250, #711‐065‐152, Jackson Immunoresearch) and streptavidin‐conjugated Alexa Fluor 488 (1/200, S11223, Invitrogen). NeuN was visualized with a CF568‐conjugated anti‐guinea pig IgG antibody (1/200, raised in donkey, #20377, Biotium). The sections were examined with a fluorescence microscope (BZ‐8000, Keyence, Osaka, Japan), and the number of labeled cells was counted in a blinded manner to the treatment.

### Data analysis and statistics

After analogue‐to‐digital conversion (PowerLab, ADInstruments), the respiratory flow signal was fed into a personal computer and the respiratory frequency and tidal volume were calculated. The baseline value was defined as the average during 30 sec before stimulation, and the response value was the average during 30 or 60 sec of stimulation. Wilcoxon's matched pair nonparametric test was used to compare baseline and response values. A two‐factor (animal x treatment) ANOVA and Tukey's post hoc test were used to compare the values among groups. GraphPad Prism software (GraphPad Software, Inc.) was used for these calculations. Data are presented as mean **± **SEM, and differences with *P* values less than 0.05 were considered significant.

## Results

### AITC caused bradypnea only when applied to the upper airway

Baseline values of respiratory frequency (WT; 231 ± 6 min^−1^, KO; 229 ± 8 min^−1^, OB; 237 ± 7 min^−1^, *n* = 7 each, *P* = 0.834) and tidal volume (WT; 0.11 ± 0.01 mL, KO; 0.11 ± 0.01 mL, OB; 0.10 ± 0.01 mL, *n* = 7 each, *P* = 0.826) were not different among the groups, indicating normal basal levels of ventilation in KO and OB animals.

**Figure 2:**
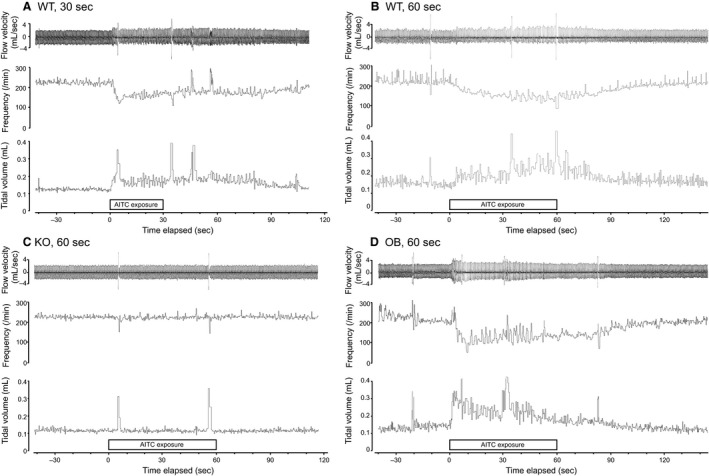
Typical trace showing allyl isothiocyanate (AITC)‐induced bradypnea. (A, B) Typical traces are shown from a wild‐type mouse, (C) TRPA1 knockout mouse, and (D) olfactory bulbectomized mouse with upper airway allyl isothiocyanate (AITC) stimulation. A record of flow velocity (upper trace), respiratory frequency (middle trace), and tidal volume (bottom trace) was calculated using PowerLab software.

**Figure 3:**
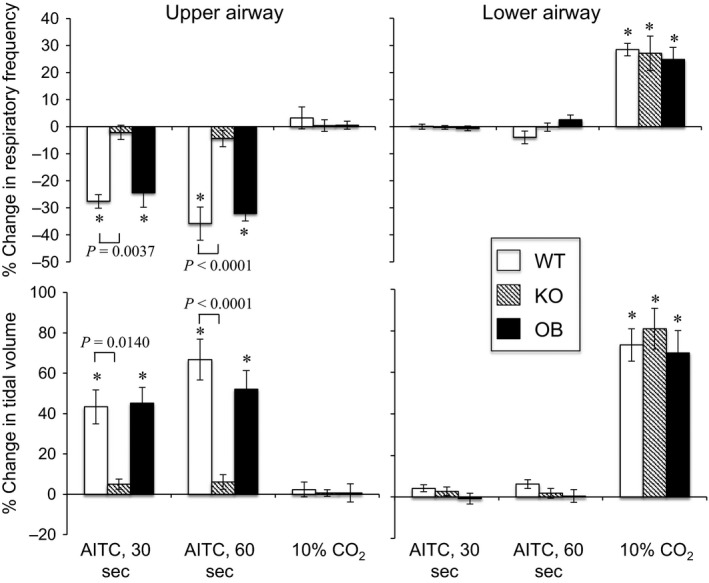
Respiratory responses to allyl isothiocyanate (AITC) vapor exposure. Upper and lower panels show changes in respiratory frequency and tidal volume, respectively. Left and right panels show results from upper and lower airway stimulation, respectively. Each column represents mean ± SEM in seven animals. * *P *<* *0.05 compared with baseline value (Wilcoxon's matched pair test). *P* values in the figure were calculated by two‐factor (animal x treatment) ANOVA and Tukey's multiple comparison test.

In the WT mouse, exposure to AITC vapor in the upper airway caused an abrupt decrease in respiratory frequency and an increase in tidal volume within 30 sec (Fig. 2A), and prolonged exposure (60 sec) further decreased and increased these values, respectively. However, the difference between 30 sec and 60 sec was not significant (Figs. 2B and 3) (*P* = 0.996 for respiratory frequency and *P* = 0.646 for tidal volume, Tukey's multiple comparisons test). These parameters rapidly returned to baseline after cessation of the stimulus. When AITC was introduced to the lower airway, however, there were no changes in respiratory frequency or tidal volume. In sharp contrast, hypercapnia caused respiratory changes (increases in frequency and tidal volume) when applied to only the lower airway.

In the KO mice, only negligible changes were observed in both upper and lower airway AITC stimulation (Figs. 2C and 3). Lower airway stimulation with hypercapnia in the KO mice caused similar changes to those in the WT mice, revealing no general abnormality in the respiratory reflex in the KO mice.

In the OB mice, the responses were almost identical to those observed in the WT mice (Figs. 2D and 3), indicating minimum contribution of the olfactory system in the AITC‐induced respiratory slowing.

In a subset of WT mice (*n* = 4) when the carrier gas was changed into 100% O_2_ from normal air (20% O_2_), AITC stimulation (60 sec) to the upper airway caused a similar decrease in respiratory frequency (−41 ± 6% in hyperoxia vs. −40 ± 6% in normoxia, *P* > 0.999) but blunted the increase in tidal volume (19 ± 2% in hyperoxia vs. 66 ± 9% in normoxia, *P* = 0.029), therefore demonstrating that observed changes in tidal volume were secondary to hypoxemia due to respiratory slowing.

### AITC activated trigeminal neurons

**Figure 4:**
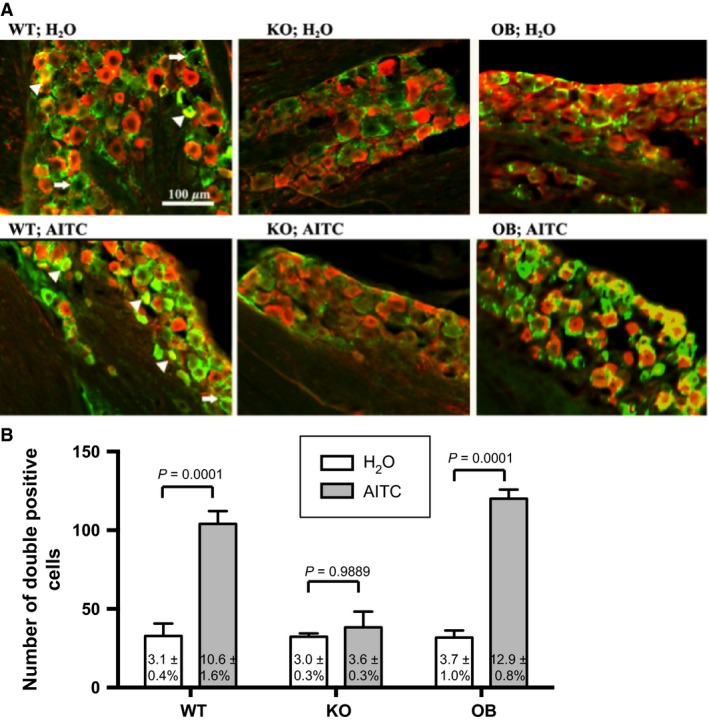
Activation of trigeminal ganglion neurons by allyl isothiocyanate (AITC) vapor exposure. (A) Representative photographs show cellular activation marker pERK (green) and neuronal marker NeuN (red) in trigeminal ganglia from six groups of mice. (B) Number of pERK and NeuN double‐positive cells in the trigeminal ganglia. Each column represents mean±SEM in four animals. Numbers at bottom of columns indicate % of pERK‐positive cells in the total NeuN‐positive cells. *P* values in the figure were calculated by two‐factor (animal x treatment) ANOVA and Tukey's multiple comparison test.

pERK‐like immunoreactivity was observed in both neurons (Fig. 4A, triangles) and satellite glial cells (Fig. 4A, arrow) in the trigeminal ganglion. To analyze neuronal activation, we counted the number of cells that were positive for NeuN and also the cells that were positive for both pERK and NeuN. Sampling bias seemed minimal because numbers of NeuN‐positive cells were not different among the groups (1022 ± 139 in WT‐H_2_O, 1033 ± 114 in WT‐AITC, 1092 ± 136 in KO‐H_2_O, 1038 ± 208 in KO‐AITC, 936 ± 104 in OB‐H_2_O, 943 ± 95 in OB‐AITC, *n* = 4 each, *P* = 0.966 for interaction, *P* = 0.653 for animal, and *P* = 0.919 for drug). As expected, inhalation of AITC for 60 s increased double‐labeled cells in the trigeminal ganglia from the WT and the OB mice but not from the KO mice (Fig. 4B), indicating that AITC activated the trigeminal neurons via TRPA1.

## Discussion

To examine the relative importance of TRPA1 in the upper and lower airways in irritant‐induced respiratory slowing, we separately stimulated the nasal cavity and the trachea/lungs with AITC and measured respiration. Nasal, but not tracheal, application of AITC vapor induced an immediate and tentative slowing of the respiratory rate in WT mice but not in TRPA1 KO mice. Olfactory bulbectomy had a minimal effect on the response in WT mice. Exposure to AITC vapor induced an activation of trigeminal neurons in WT and OB mice but not in TRPA1 KO mice. These data clearly showed for the first time that nasal, and possibly trigeminal, TRPA1 plays an essential role in irritant‐induced bradypnea. Participation of TRPA1 in the trachea/lungs and olfactory bulb in irritant‐induced bradypnea seemed to be minimal.

Different respiratory outcomes between nasal and tracheal challenges of the same drug have already been reported (Chou et al. 2008). In anesthetized guinea pigs, hypertonic saline (6% NaCl) and citric acid induced bradypnea when applied to the nasal cavity, but only had a negligible effect when applied to the trachea. Capsaicin, a TRPV1 agonist, decreased respiratory frequency with nasal application, but increased it with tracheal application. Although these drugs were applied in liquid form, the results are in line with our current report showing different respiratory effects depending on location of application using AITC vapor.

Although TRPV1 involvement in upper airway‐initiated bradypnea (Chou et al. 2008) may infer TRPA1 participation due to the pair often being coexpressed, only 60–70% of TRPV1‐positive cells in the trigeminal ganglion also contain TRPA1 (Kobayashi et al. 2005). Therefore, activation of the entire TRPV1 population by capsaicin may have a different effect because 30–40% of TRPV1‐positive cells do not contain TRPA1. Also, TRPV1 is well characterized as a heat‐sensing channel, and although TRPA1's role in cold‐sensing is controversial (de Oliveira et al. 2014), its function in regard to temperature is most likely disparate. Thus, capsaicin application would not necessarily produce the same result as TRPA1 activation. To our knowledge, ours is the first report comparing the relative importance of TRPA1 activation in the upper and lower airways (trigeminal/vagal nerves) on respiratory function in the same animal.

We expected that lower airway stimulation would affect respiratory frequency at least to some extent because vagal afferent neurons innervating trachea in the nodose ganglion express TRPA1 (Bessac et al. 2008; Takahashi et al. 2011) and stimulation of tracheo‐bronchial irritant receptors and lung C‐fiber receptors induces gasping and apnea (Coleridge and Coleridge 1986). Other researchers have also reported a possibility of vagal contribution to irritant‐induced bradypnea (Prabhakar et al. 1986; Kou et al. 1995; Wang et al. 1996; Nassenstein et al. 2008). However, in the present study, responses to AITC were negligible when it was introduced to the lower airway in the WT mice. This apparent discrepancy may be explained in the following two ways. First, TRPA1 expression in tracheal/lung afferents may be lower than in nasal afferents. This possibility is difficult to consider, however, because all the available evidence only shows the existence of TRPA1 protein or mRNA in ganglionic cell bodies and not in nerve terminals where it could be easily activated by irritants. Second, TRPA1‐containing nodose ganglionic neurons may be different from those expressing tracheo‐bronchial irritant receptors or lung C‐fiber receptors. We do not intend to deny a role of TRPA1 in the lower airway. Rather, we speculate one possible role of TRPA1 in the lower airway may be to induce defensive responses other than bradypnea such as coughing and/or inflammation (Bautista et al. 2006; Viana 2016). This hypothesis should be further examined by experimentation with a different setting from the current study because anesthesia was necessary for cannula insertion and coughing and sneezing responses cannot be observed under these conditions (Douglas 2000). Guinea pigs are the only known experimental animal species that show coughing‐like behavior in an anesthetized condition (Chou et al. 2008).

OB mice showed similar bradypnea and trigeminal nerve activation as WT mice. Again, we do not intend to deny a role for olfactory nerve defensive responses to airborne threats. Olfactory nerves may protect animals from airborne threats by employing a defensive output strategy other than bradypnea.

In the present study, we successfully showed activation of trigeminal ganglion neurons by AITC vapor using pERK immunostaining. ERK phosphorylation may occur very rapidly compared to the synthesis of certain proteins, such as c‐Fos, which are commonly used to detect neuronal activation (Iwashita et al. 2013; Antoine et al. 2014). Therefore, pERK immunostaining is a suitable histological tool for detecting rapid neuronal activation associated with transient responses such as AITC‐induced bradypnea. Nevertheless, we should keep in mind that pERK is not simply a rapid version of c‐Fos as the mechanism of ERK phosphorylation is different from that of c‐Fos production and diverse in itself (Grewal et al. 1999). There are actually abundant reports showing an increase in c‐Fos expression in the spinal trigeminal nucleus, the second‐order relay neurons of the trigeminal system, but not in the trigeminal ganglion neurons themselves upon sensory stimulation (Anton et al. 1991; Groneberg et al. 2001; Yonemitsu et al. 2013).

In conclusion, trigeminal, but not vagal or olfactory, TRPA1 is essential for irritant‐induced bradypnea. We have previously shown that nasal TRPA1 is essential for environmental chemical‐induced avoidance behavior and arousal from sleep (Yonemitsu et al. 2013). Taken together, nasal TRPA1 seems to be a frontline alarm against environmental threats.

## Conflict of Interest

None declared.
